# Membrane Potential-Dependent Uptake of Cationic Oligoimidazolium Mediates Bacterial DNA Damage and Death

**DOI:** 10.1128/aac.00355-23

**Published:** 2023-05-01

**Authors:** Melvin Yong, Zhi Y. Kok, Chong H. Koh, Wenbin Zhong, Justin TY. Ng, Yuguang Mu, Mary B. Chan-Park, Yunn-Hwen Gan

**Affiliations:** a Department of Biochemistry, Infectious Diseases Translational Research Programme, Yong Loo Lin School of Medicine, National University of Singapore, Singapore, Singapore; b School of Chemistry, Chemical Engineering and Biotechnology, Nanyang Technological University, Singapore, Singapore; c Centre for Antimicrobial Bioengineering, Nanyang Technological University, Singapore, Singapore; d School of Biological Sciences, Nanyang Technological University, Singapore, Singapore

**Keywords:** DNA damage, cationic, enteric bacteria, experimental therapeutics, imidazolium, membrane potential

## Abstract

The treatment of bacterial infections is becoming increasingly challenging with the emergence of antimicrobial resistance. Thus, the development of antimicrobials with novel mechanisms of action is much needed. Previously, we designed several cationic main-chain imidazolium compounds and identified the polyimidazolium PIM1 as a potent antibacterial against a wide panel of multidrug-resistant nosocomial pathogens, and it had relatively low toxicity against mammalian epithelial cells. However, little is known about the mechanism of action of PIM1. Using an oligomeric version of PIM1 with precisely six repeating units (OIM1-6) to control for consistency, we showed that OIM1-6 relies on an intact membrane potential for entry into the bacterial cytoplasm, as resistant mutants to OIM1-6 have mutations in their electron transport chains. These mutants demonstrate reduced uptake of the compound, which can be circumvented through the addition of a sub-MIC dose of colistin. Once taken up intracellularly, OIM1-6 exerts double-stranded DNA breaks. Its potency and ability to kill represents a promising class of drugs that can be combined with membrane-penetrating drugs to potentiate activity and hedge against the rise of resistant mutants. In summary, we discovered that cationic antimicrobial OIM1-6 exhibits an antimicrobial property that is dissimilar to the conventional cationic antimicrobial compounds. Its killing mechanism does not involve membrane disruption but instead depends on the membrane potential for uptake into bacterial cells so that it can exert its antibacterial effect intracellularly.

## INTRODUCTION

The rise of antimicrobial resistance (AMR) has undermined the effectiveness of conventional antibiotic treatments. We have witnessed the escalating prevalence of multidrug resistant bacteria over the past few decades, including examples such as the six nosocomial pathogens that are collectively known as the ESKAPE pathogens on the World Health Organization’s critical priority 1 list of bacterial pathogens in need of new antibiotics (1, 2). Thus, AMR poses a grave threat to global human health and health systems. Drug resistance, as well as the paucity of novel and effective antibiotics, has accelerated the progression toward a postantibiotic era, meaning an era that is deprived of therapeutic options against resistant bacterial infections. Thus, new antimicrobials with novel mechanisms of action are greatly needed.

Antimicrobial peptides (AMPs) are structurally diverse peptides that have antibacterial properties (3). AMPs are promising antibacterials because they are less prone to resistance development due to their membrane active mechanism of action and their pharmacodynamic properties differing from those of conventional antibiotics (4). However, the production of AMPs is often costly (5), and the toxicity, bioavailability, and stability of some AMPs is a concern (6). As such, synthetic mimics of AMPs are interesting alternatives that could capture their properties (hydrophilic groups and hydrophobic moieties) while remaining inexpensive, easier to synthesize, and highly stable (7). We have previously designed a series of the synthetic mimics, which are known as the cationic polyimidazolium polymers (PIMs) (8). We successfully identified PIM1 as a bactericidal antimicrobial compound that is effective against a broad spectrum of drug-resistant Gram-positive and Gram-negative clinical pathogens with low MICs that range from 1 to 8 μg/mL (8). The half maximal inhibitory concentration (IC_50_) against the epithelial cell lines that were tested were similar, if not better, than those of the clinically used cationic antimicrobial peptides (i.e., colistin or polymyxin B). These properties indicate that PIM1 may be a promising antimicrobial compound. We have also observed that the MIC of PIM1 is influenced by the membrane potential (ΔΨ), as dissipating the ΔΨ with valinomycin causes an upward MIC shift (8). However, its precise mechanism remains elusive, and it is unclear whether it exerts its antimicrobial effect on the membrane or intracellularly. Here, we utilized the oligomer of PIM1 with six repeating units, OIM1-6, for our mechanistic study so as to gain important insights regarding its mechanism of killing.

## RESULTS

### The oligomer OIM1-6 retains the efficacy of PIM1.

**FIG 1:**
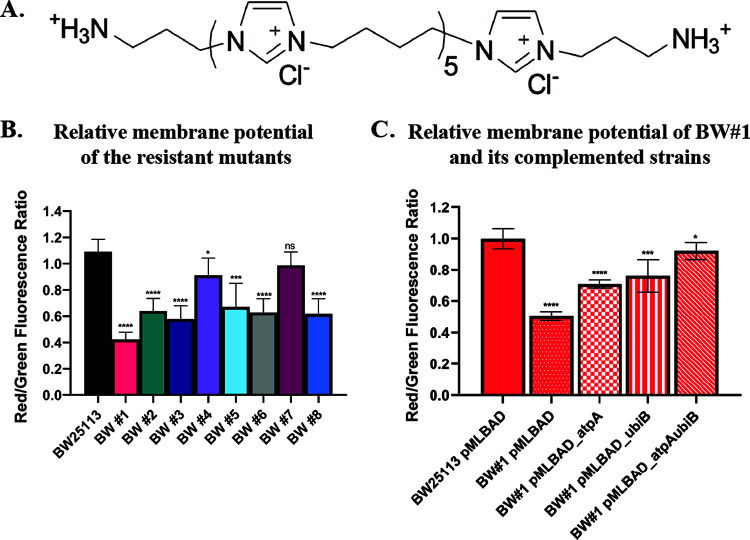
Relative membrane potential of the E. coli BW25113 resistant mutants. (A) Chemical structure of OIM1-6. (B) Membrane potential measurement using DiOC_2_(3) voltage sensitive dye on E. coli BW25113 wild-type and the OIM1-6-resistant mutants. (C) The membrane potential of the BW#1 resistant mutant and the various complemented strains. The data shown are from three independent experiments. Error bars denote the standard deviation. The statistical significance of the differences between the wild-type BW25113 and the other groups was assessed using unpaired *t* tests: ns, *P* > 0.05; *, *P* ≤ 0.05; **, *P* ≤ 0.01; ***, *P* ≤ 0.001; ****, *P* ≤ 0.0001.

PIM1 is a polymer with various repeating imidazolium subunits (8). As such, it does not have a defined molecular weight and is therefore not ideal for our mechanistic study because of the heterogeneity. Therefore, we designed an oligomer of PIM1 with six repeating imidazolium subunits, namely, OIM1-6 (Fig. 1A), which has a defined molecular weight of 1,049.34 g/mol. We performed MIC tests on *Enterobacteriaceae* strains (i.e., Escherichia coli and Klebsiella pneumoniae), and we found the MIC of OIM1-6 (Table 1) to be 4 μg/mL, which is comparable to the previously published MIC of PIM1 against E. coli and K. pneumoniae strains (8). Similar to PIM1, OIM1-6 is also bactericidal against E. coli. Time-kill assays revealed that OIM1-6 inhibited E. coli growth at 1× and 2× MIC and had a bactericidal effect at 4× MIC (Fig. S1A and B). Similar to PIM1, OIM1-6 does not cause membrane disruption (Fig. S2). The resemblance of OIM1-6 to PIM1 in terms of efficacy renders it a good substitute for PIM1 in our study.

**TABLE 1:** MIC of OIM1-6

Bacterial strain	Minimum inhibitory concentration of OIM1-6 (μg/mL)
K. pneumoniae SGH10^*a*^	4
K. pneumoniae BAK085^*a*^	4
K. pneumoniae M7^*a*^	4
E. coli MG1655^*a*^	4
E. coli BW25113	4
E. coli BW25113::pMLBAD^*b*^	4
E. coli BW#1	128
E. coli BW#1::pMLBAD^*b*^	128
E. coli BW#1::pMLBAD_*atpA*^*b*^	32
E. coli BW#1::pMLBAD_*ubiB*^*b*^	16
E. coli BW#1::pMLBAD_*atpA_ubiB*^*b*^	4
E. coli BW#2	16
E. coli BW#2::pMLBAD^*b*^	16
E. coli BW#2::pMLBAD_*ubiX*^*b*^	4
E. coli BW#3	32
E. coli BW#3::pMLBAD^*b*^	32
E. coli BW#3::pMLBAD_*hemA*^*b*^	4
E. coli BW#4	16
E. coli BW#5	32
E. coli BW#6	32
E. coli BW#7	16
E. coli BW#8	32
E. coli BW25113Δ*uvrD* (KEIO name: JW3786)	8
E. coli BW25113Δ*rfaC* (KEIO name: JW3596)	8

a The MIC_PIM1_ for SGH10, BAK085, M7, and MG1655 is 8 μg/mL.

b The MIC values of these strains are measured in MHB supplemented with 0.2% arabinose.

### Mutations on electron transport chain-related genes lead to the lower susceptibility of E. coli to OIM1-6.

In our previous experiment with PIM1, we learned that PIM1 potency is influenced by the membrane potential (ΔΨ) (8). However, the mechanism of action remains undefined. To explore this via a genetics approach, we screened for spontaneous OIM1-6-resistant mutants, using the E. coli K-12 strain BW25113. We successfully isolated 8 clones (BW#1 to BW#8), with the BW#1 mutant exhibiting a 32-fold increase in MIC, whereas the rest developed a more modest 4 to 8-fold MIC increase (Table 1). The whole-genome sequencing data of these clones reveal mutations mainly in the electron transport chain (ETC)-related genes (e.g., ATP synthase, ubiquinone, heme, cytochrome oxidase) (Table 2). Notably, the BW#1 mutant has two mutations in its ETC genes (*atpA* and *ubiB*), whereas the rest of the mutants predominantly have one mutation in their ETC genes. We measured the growth rates of BW#1, BW#2, and BW#3, and observed that these mutants exhibited growth defects, compared to their wild-type counterparts (Fig. S3A and B). Complementation using the wild-type *atpA* gene on the pMLBAD plasmid with an arabinose-inducible promoter (pMLBAD_*atpA*) into BW#1 resulted in the partial restoration of susceptibility to OIM1-6 from 128 μg/mL to 32 μg/mL (Table 1). Similarly, complementation using pMLBAD_*ubiB* restored the MIC to 16 μg/mL. Furthermore, the complementation of both wild-type genes (pMLBAD_*atpA_ubiB*) into the BW#1 mutant fully restored its susceptibility to the level observed for the wild-type (MIC of 4 μg/mL). We also performed complementation on the BW#2 and BW#3 mutants with pMLBAD_*ubiX* and pMLBAD_*hemA,* respectively, and found that their resistance to OIM1-6 was abolished after complementation (Table 1). In addition, the growth rates of the mutants were also restored with the complementation (Fig. S3C and D). This led us to consider whether a lower growth rate is a probable factor that contributes to OIM1-6 resistance. Then, we examined the MIC of OIM1-6 against other non-ETC related mutants (helicase deletion mutant BW25113Δ*uvrD* and LPS biosynthesis mutant BW25113Δ*rfaC*) with growth defects (Fig. S4). However, we observed only up to a 2-fold increase in MIC (Table 1), suggesting that a slow growing phenotype is not accountable for higher resistance against OIM1-6. These results demonstrate that there is a factor, other than the slower growth, that is responsible for the OIM1-6 resistance in these ETC mutants.

**TABLE 2:** Genomic profile of the OIM1-6-resistant mutants

Bacterial strains	Mutations identified (compared against Ref Seq NZ_CP009273.1)	Remarks
BW25113	No mutation	-^*b*^
BW#1	*atpA ←* Δ309 bp (971 – 1279/1542 nt)	Frameshift mutation
	*ubiB →* N328K (AAC→AAA)^*c*^	Missense mutation PROVEAN^*a*^ score (−5.488) = Deleterious SIFT^*a*^ score (0.00) = Intolerant
BW#2	*ubiX ←* P86H (CCC→CAC)	Missense mutation PROVEAN score (−8.950) = Deleterious SIFT score (0.00) = Intolerant
BW#3	*hemA →* G119C (GGT→TGT)	Missense mutation PROVEAN score (−8.789) = Deleterious SIFT score (0.00) = Intolerant
BW#4	Unassigned new junction evidence: *cydB* and *cydB/cydX*	-
BW#5	*hemG →* Δ1 bp (449/546 nt)	Frameshift mutation
BW#6	*hemL ←* +T (454/1281 nt)	Frameshift mutation
BW#7	*cydB →* 2 bp→AC (993-994/1140 nt)	Frameshift mutation
	*icd →*H366H (CAC→CAT)	Missense mutation SIFT score (0.18) = Tolerated
	*icd →* T370T (ACC→ACT)	
	*icd →* L375M (TTA→CTG)	
	*icd →* L375M (TTA→CTG)	
BW#8	*[fhuD], fhuB, hemL, [clcA]* (Δ5,237 bp)	Multiple gene deletion
	*pgaC ←* K426K (AAG→AAA)	Silent mutation

a The impact of a single nucleotide polymorphism is assessed using the Protein Variation Effect Analyzer (PROVEAN) (35), with a score of <−2.5 being predicted to be deleterious (or neutral, otherwise), and/or Sorting Intolerant from Tolerant (SIFT) (36), with a score of <0.05 being predicted to be intolerant (or tolerant, otherwise).

b The dash symbol for BW25113 means that there are no remarks because there is no mutation found when compared to the reference sequence that was published. Similarly, the dash symbol for BW#4 indicates that there are no remarks because the mutation found is a new junction in the intergenic region that cannot be assigned to a specific mutation.

c Any letters that are underlined represent changes in the nucleotide sequence. For instance, if C and A at the third position are underlined and the sequence is AAC → AAA, this means that the nucleotide C has been changed to A.

### Resistance to OIM1-6 is associated with lower membrane potential and drug uptake.

Mutations in the ETC genes are known to increase the resistance against aminoglycosides via the decreased membrane potential affecting their uptake (9–11). Using 3,3′-diethyloxacarbocyanine iodide (DiOC_2_(3)) voltage-sensitive dye, we found lowered membrane potential in the resistant mutants, compared to the wild-type strain (Fig. 1B). In fact, these mutants were also resistant to membrane potential-dependent antibiotics (gentamicin) (Table S2). We also examined the membrane potential of the complemented BW#1 mutant and found that complementation with a single copy of the wild-type gene (i.e., either *atpA* or *ubiB*) led to the intermediate restoration of the membrane potential, whereas the complementation of both wild-type copies led to restoration that was closer to the level observed for the wild-type (Fig. 1C). Similarly, complementation with the respective wild-type gene also restored the membrane potential of BW#2 and BW#3 (Fig. S5). The restoration of the membrane potential is correlated with the decrease in MIC (Table 3). Taken together, these data suggest that the diminished membrane potential in the mutants is a likely factor that contributes to the resistance phenotype.

**TABLE 3:** MIC of OIM1-6 against E. coli in the absence and presence of colistin

Bacterial strain	Minimum inhibitory concentration (μg/mL) of OIM1-6			
	Without colistin	0.125× MIC colistin	0.25× MIC colistin	0.5× MIC colistin
E. coli MG1655	4	2	1	0.5
E. coli BW25113	4	2	1	0.5
E. coli BW#1	128	64	2	0.5
E. coli BW#2	16	8	1	0.5
E. coli BW#3	32	16	1	0.5

**FIG 2:**
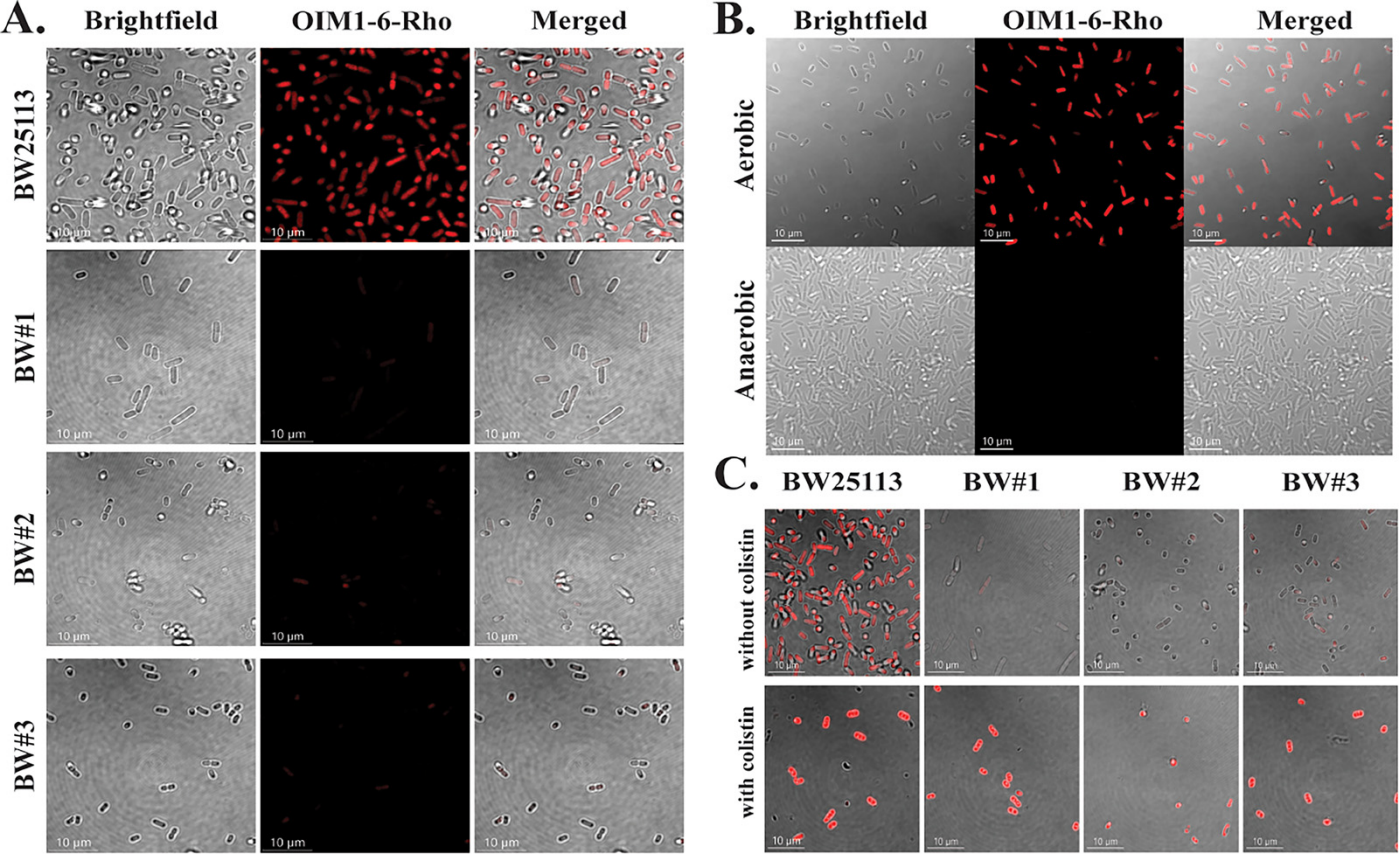
Intracellular uptake of OIM1-6-Rho in E. coli. (A) Representative confocal images of E. coli BW25113, BW#1, BW#2, and BW#3 treated with 4 μg/mL OIM1-6-Rho for 1 h in MHB at 37°C. (B) Representative confocal images of E. coli MG1655 treated with 4 μg/mL OIM1-6-Rho for 1 h in MHB at 37°C, under aerobic and anaerobic growth conditions (anaerobic chamber). (C) Representative confocal images showing OIM1-6-Rho (4 μg/mL) uptake after 1 h of treatment in the absence and presence of 0.5× MIC colistin.

To determine whether the resistant mutants exhibit lower OIM1-6 uptake relative to wild-type E. coli, we synthesized OIM1-6 labeled with rhodamine (OIM1-6-Rho [8]) for fluorescence imaging. The MIC of OIM1-6-Rho is comparable to that of untagged OIM1-6 (Table S3), indicating that the fluorescence tag on the compound did not diminish its activity. Using confocal microscopy and flow cytometry, we found lower OIM1-6-Rho levels in the selected resistant mutants (BW#1, BW#2, and BW#3) compared to the wild-type strain (Fig. 2A; Fig. S6A), suggesting a correlation between compound activity and drug uptake. We examined drug uptake under anaerobic growth conditions, which were known to result in low membrane potential. As expected, the drug uptake in wild-type E. coli grown anaerobically is markedly reduced, compared to its aerobically grown counterpart (Fig. 2B; Fig. S6B). To ensure that drug uptake was not influenced by the rhodamine tag, we examined the uptake using OIM1-6 labeled with fluorescein (OIM1-6-FITC). The results (Fig. S7) are consistent with those that were obtained by using OIM1-6-Rho, thereby showing that the differential uptake was unlikely to have been influenced by the rhodamine tag. Furthermore, we found that the reduction in drug uptake also leads to a 16-fold increase in MIC when compared between anaerobically and aerobically grown E. coli (Table S4). We also tested the MIC of the resistant mutants grown anaerobically (Table S4), and found that MICs of the mutants and the wild-type are comparable under anaerobic growth conditions. These findings support the idea that membrane potential is a major factor that influences OIM1-6 susceptibility.

As we observed intracellular uptake to be an important factor for OIM1-6 potency, we then challenged the resistant mutants with both colistin and OIM1-6 to examine whether forcing the uptake would abrogate the resistance. Colistin disrupts the outer membrane by interacting with lipopolysaccharides (LPS) and displaces the cationic inter-LPS bridges, thereby leading to damage to the cytoplasmic membrane (12). Cotreatment with colistin has been shown to enhance the potency of antibiotics with intracellular mode of action but not those with an extracellular target, such as the cell wall (13). We found that cotreatment with a subinhibitory concentration of colistin successfully restored the sensitivity of the resistant mutants to OIM1-6 to a level that was comparable to that of the wild-type E. coli (Table 3). This was also reflected by the enhanced intracellular uptake (Fig. 2C). Therefore, the restoration of intracellular uptake in the presence of colistin is accountable for the restoration of OIM1-6 potency against the resistant mutants.

### OIM1-6 binds DNA intracellularly to cause dsDNA breaks.

**FIG 3:**
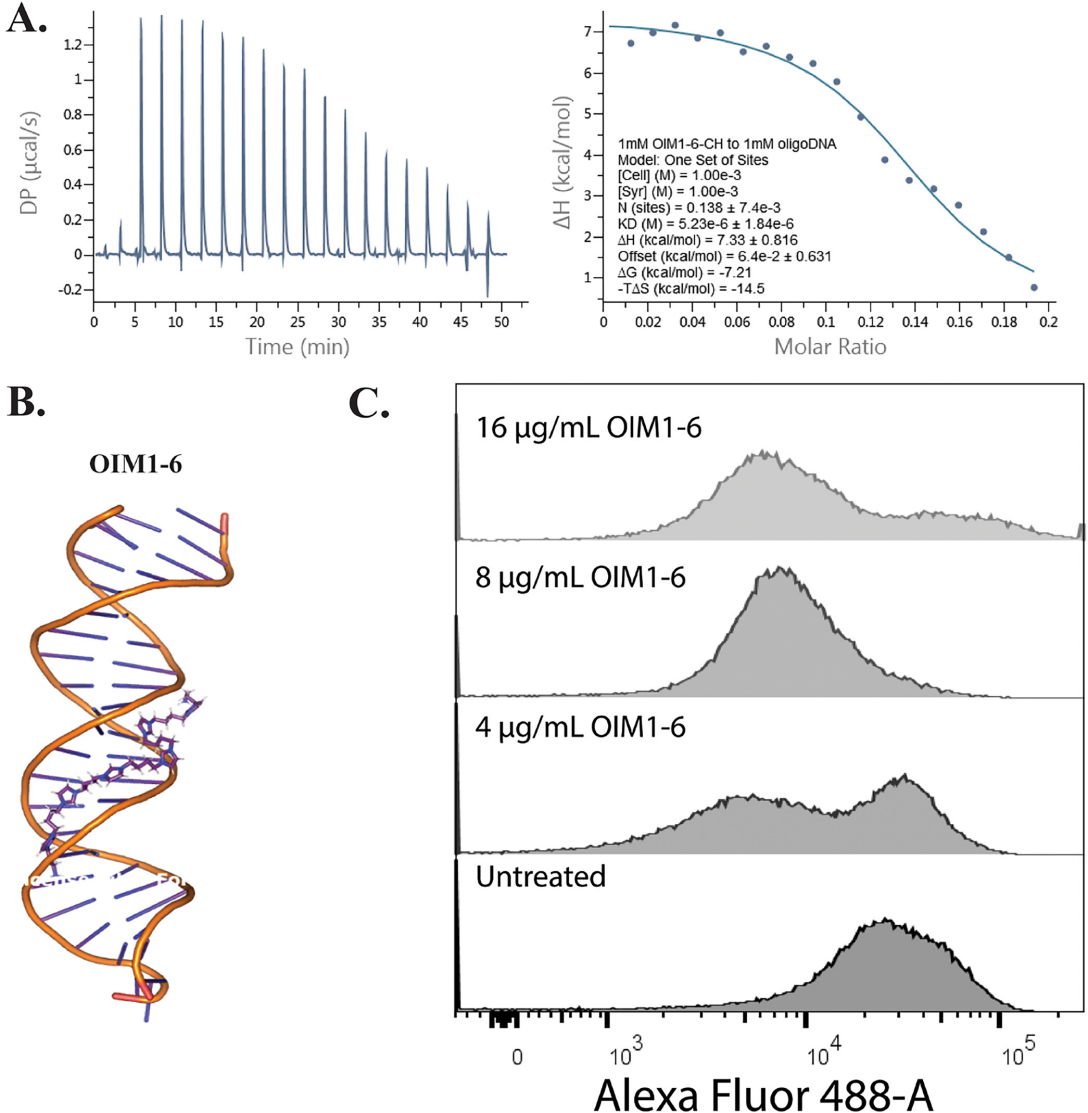
DNA binding properties of OIM1-6. (A) Representative isothermal titration calorimetry (ITC) profiles of OIM1-6 binding to oligonucleotides. The titration for the interaction between OIM1-6 and 52 bp oligonucleotides was performed in 5 mM Tris (pH 7.4) and 15 mM NaCl at 25°C. (B) *In silico* molecular simulation, showing the binding of OIM1-6 to the minor groove of DNA. (C) Representative flow cytometry analysis of the picogreen signal in OIM1-6-treated E. coli MG1655, relative to untreated cells. OIM1-6 was treated at 4, 8, and 16 μg/mL for 1 h in MHB at 37°C.

As our previous results indicate that OIM1-6 enters the bacterial cytoplasm, it is possible that cationic OIM1-6 interacts with negatively charged intracellular macromolecules such as DNA. Through an *in vitro* gel retardation assay, we found that OIM1-6 retarded DNA band migration (Fig. S8). We also tested its ability to displace picogreen *in vitro*. Picogreen is a DNA binding dye that selectively binds to dsDNA and displays high fluorescence enhancement when bound to DNA but virtually no fluorescence in its unbound form, thereby facilitating a good noise-to-signal ratio (14). Similar to the gel retardation results, we observed that OIM1-6 could displace picogreen binding in a concentration-dependent manner (Fig. S9). Interestingly, the isothermal titration calorimetry (ITC) results of OIM1-6 with DNA (Fig. 3A) show a positive enthalpy value and a positive entropy change (ΔS), implying that the interaction is likely to be driven not electrostatically, but by a predominantly hydrophobic effect. The results of an *in silico* molecular simulation indicate that OIM1-6 binds to the minor groove of the DNA, thereby leading to the release of bound water (Fig. 3B) as reported in distamycin-DNA or Hoechst-DNA interactions (15, 16). To determine whether such DNA binding takes place intracellularly, we measured the signal of picogreen in untreated and OIM1-6-treated cells via a flow cytometric analysis (Fig. 3C). We observed a reduction of the picogreen signal in the OIM1-6-treated cells relative to the untreated control. Although there is the possibility that OIM1-6 affects the membrane structure and therefore affects the picogreen signal, it is more likely due to the binding of OIM1-6 to the DNA. This binding displaces picogreen and leads to a reduction of the intracellular picogreen signal for the OIM1-6-treated cells. These results are in line with the ability of OIM1-6 to directly bind DNA (Fig. S8 and S9).

**FIG 4:**
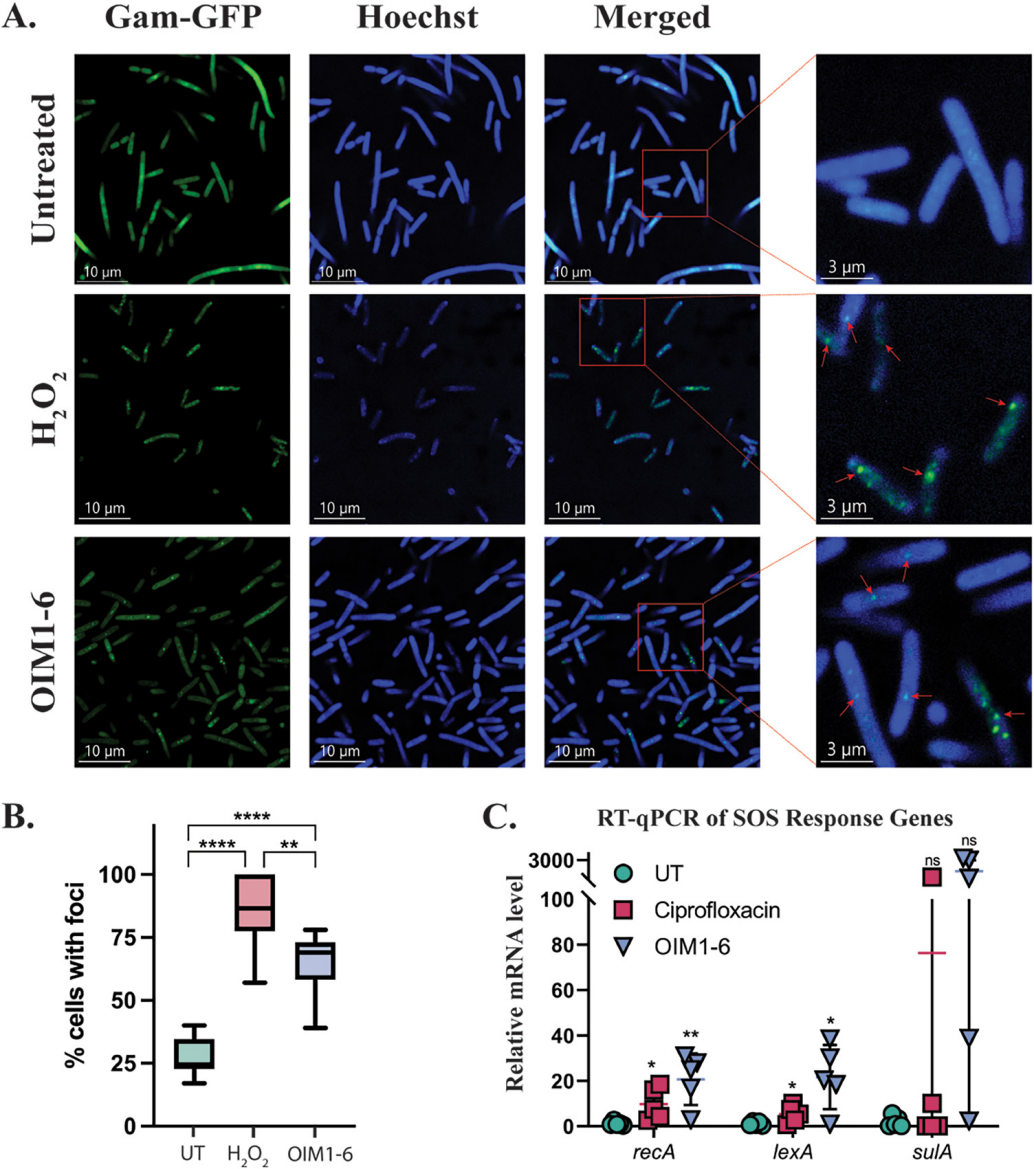
OIM1-6 treatment leads to double-stranded DNA breaks and the induction of the expression of SOS response genes in E. coli. (A) Representative confocal images of the E. coli MG1655 SMR14334 Gam-GFP strain without any treatment (negative control), treated with 10 mM hydrogen peroxide (positive control), or treated with 1× MIC OIM1-6 for 2 h in MHB at 37°C. The green color represents the Gam-GFP signal, and the blue color represents the DNA dye Hoechst. Foci formation indicates double-stranded DNA breaks, and these are marked by the red arrows in the zoomed-in images. (B) Percentage of cells with Gam-GFP foci for the three different treatment groups (Untreated [UT]; 10 mM hydrogen peroxide and 1× MIC OIM1-6), with at least 100 bacterial cells having been quantification for each condition. (C) Representative RT-qPCR data of the SOS response genes (*recA*, *lexA*, and *sulA*) in E. coli MG1655 treated with 2× MIC OIM1-6 or ciprofloxacin for 2 h. Error bars denote the standard deviation. ns, *P* > 0.05; *, *P* ≤ 0.05; **, *P* ≤ 0.01 (unpaired *t* test).

The DNA binding properties of OIM1-6 suggest that the drug could cause DNA damage. To examine whether OIM1-6 treatment causes intracellular DNA damage, we utilized the E. coli Gam-GFP strain (17), which is engineered for the detection of double-stranded DNA (dsDNA) breaks that manifest in the form of GFP foci under confocal microscopy. Indeed, we observed the formation of multiple foci (percentage of cells with foci: 85.5 ± 13.9) for cells treated with hydrogen peroxide (18) as a positive-control (Fig. 4A and B). Interestingly, the OIM1-6-treated cells exhibited more foci (percentage of cells with foci: 65.3 ± 11.5), compared to the untreated cells (percentage of cells with foci: 27.5 ± 7.3) (Fig. 4B). In line with the observed dsDNA break, the DNA damage response genes *recA* and *lexA* were found to be significantly induced upon OIM1-6 treatment, whereas the induction of *sulA* did not reach statistical significance (Fig. 4C). Taken together, these results demonstrate that OIM1-6 enters the bacterial cytoplasm, leading to DNA binding and dsDNA breaks triggering the SOS response.

## DISCUSSION

We had designed cationic polyimidazolium compounds (8), such as PIM1, as potential antibacterial agents. Unlike other cationic antimicrobials, for which the mechanism of action involves bacterial membrane disruption (19), PIM1 does not disrupt the transmembrane electrochemical gradient or membrane integrity (8). Our previous study reported the dependency of the proton motive force for the activity of PIM1 via the use of chemicals such as valinomycin and nigericin to distinguish between the contributions of ΔΨ and ΔpH (8). One limitation is the potential off-target effects of these drugs. Employing a genetic approach via the screening of E. coli OIM1-6-resistant mutants, we found that the potency of the oligomeric drug is associated with the membrane potential-dependent uptake, which is driven by the ETC. This is the first time cationic polyimidazolium compounds have been shown to be dependent on the membrane potential for uptake. One class of antibiotics that is also dependent on the membrane potential for uptake is the aminoglycosides (10). Despite nearly 80 years having passed since the discovery of the first aminoglycoside, the intracellular uptake of aminoglycosides is still not well-understood, with conflicting data reported. However, it is generally thought to occur in three stages (20, 21). The first stage involves the electrostatic interaction between the cationic moieties of aminoglycosides, the negatively charged lipopolysaccharides in the outer membrane, and the subsequent ionic association with the inner membrane (9, 22). The second stage is called the energy-dependent phase I (EDP-I), and it involves a low rate of energy-dependent uptake that is reliant on a threshold of membrane potential and the concentration of the aminoglycoside (9). This stage can be blocked by respiratory inhibitors, such as oxidative phosphorylation or electron transport inhibitors (23, 24). The third stage, which is known as EDP-II, involves accelerated energy-dependent uptake that utilizes the energy that is generated from electron transport or ATP hydrolysis (22). More recently, a membrane voltage-induced bactericidal model was also proposed (25). Whereas the OIM1-6-resistant mutants are less susceptible to an aminoglycoside (gentamicin) due to their reduced membrane potential, it is unclear whether the uptake of both drugs occurs in a similar manner. What is clear from our genetic analyses is that the drug is not acting specifically on only one target. It interacts with components on the ETC for entry. Upon entry, it binds to DNA to mediate DNA damage.

We found that the binding of OIM1-6 to DNA causes dsDNA breaks. The DNA binding properties have been elucidated in a few cationic AMPs such as LL-37 (26) and Frenatin 2.3S (27), as well as in cationic synthetic polymers such as polyhexamethylene biguanide (28) and oligoimidine (29). However, as these antimicrobials are membrane-active with membrane disruptions as their primary mechanism, the nucleic acid binding is considered to be a secondary effect. In contrast, the DNA binding mechanism would be more relevant as the primary mechanism for nonlytic antimicrobials, such as indolicidin (30), as well as for our cationic oligomer OIM1-6. As extensive DNA damage is observed within a short time frame, we believe that this is one way in which the drug exerts its toxicity in bacterial cells. However, its DNA binding properties may be nonspecific due to the physicochemical properties of the drug. This may explain our difficulty to generate high resistance and the fact that all of the resistant mutants obtained are related to uptake. The disadvantage of such an antibacterial with an intracellular mode of action is that its efficacy relies on its capability of translocating into the bacterial cytoplasm. This means that the potency of OIM1-6 can be undermined by factors that lead to diminished membrane potential and uptake, such as bacterial growth under low oxygen tension or bacteria with altered electron transport. However, as shown by our results with colistin, a combinatorial treatment with a subinhibitory concentration of a membrane-permeabilizing compound could mitigate this and potentially lead to an effective treatment.

In summary, we discovered that the cationic antimicrobial OIM1-6 exhibits an antimicrobial property that is dissimilar to that of conventional cationic antimicrobial compounds. Its killing mechanism does not involve membrane disruption but instead depends on the membrane potential for intracellular uptake into bacterial cells, resulting in DNA damage.

## MATERIALS AND METHODS

### Bacterial strains and growth conditions.

E. coli (MG1655 and BW25113 [31]) and K. pneumoniae (SGH10, M7, and BAK085) were maintained on lysogeny agar (LA) at 37°C. Unless specified otherwise, they were propagated for experimental usages in Mueller-Hinton Broth (MHB) at 37°C with shaking at 150 rpm.

### MIC assay.

The MICs of the drugs were determined via broth microdilution (8, 32). Bacterial colonies were taken from freshly streaked LA for overnight growth in MHB. The overnight cultures were subcultured at a 1:100 dilution to the mid-log phase in MHB, and they were then diluted to a bacterial density of 10^6^ CFU/mL in MHB. The bacterial suspensions were then added into 96-well plates containing a gradient of tested drug concentrations at 2-fold dilutions, including a drug-free positive control and a medium-only negative control. The plates were then statically incubated at 37°C for 18 h before the optical density at 600 nm (OD_600_) was measured.

### Screening for resistant mutants and whole-genome data analysis.

We adopted the mutation rate assay (33) with modifications for screening and isolating the resistant mutants. Briefly, a single colony of the E. coli BW25113 wild-type strain was inoculated in MHB and grown overnight at 37°C with shaking at 150 rpm. The overnight culture was then subcultured into 8 tubes containing MHB at 1,000 CFU/mL and grown to saturation. The bacterial culture from each tube was then diluted to 10^6^ CFU/mL, and 100 μL were transferred to each row of a 96-well plate containing 100 μL OIM1-6 at a final concentration of 16 μg/mL. The plate was incubated for 48 h at 37°C. Wells with positive bacterial growth were streaked out on LA, and a single colony was subjected to MIC testing and whole-genome sequencing. Illumina whole-genome sequencing was performed on the wild-type bacteria and the resistant mutants via Novogene AIT. The genome files (FASTQ) were analyzed using Breseq (34) for potential mutations. Single nucleotide polymorphisms were further analyzed by PROVEAN (35) and/or SIFT (36) for the prediction of their impact on protein function. The sequence data can be found in the NCBI database under BioProject PRJNA941003.

### Complementation of the wild-type gene in resistant mutants.

The PstI and EcoRI linearized arabinose-inducible plasmid pMLBAD (37) was assembled with a PCR product (gene of interest) using NEBuilder HiFi DNA Assembly, following the manufacturer’s protocol. The PCR products were amplified using the primers listed in Table S1. The assembled product was added to TaKaRa Stellar competent cells in an Eppendorf tube with 30 min of incubation on ice. The cells were subsequently subjected to a heat shock by placing them on a water bath at 42°C for 45 secs and transferring them to ice for 2 min of incubation. After 1 h of recovery at 37°C in lysogeny broth, the bacteria were plated on LA containing 15 μg/mL trimethoprim for overnight growth at 37°C. Colonies were screened to determine the presence of the gene of interest in pMLBAD via agarose gel electrophoresis and sequencing (1st Base). The validated pMLBAD plasmids containing the gene of interest were electroporated into the respective mutant strains using MicroPulser electroporation cuvettes (0.2 cm gap) (Bio-Rad) and a MicroPulser Electroporator (Bio-Rad). For the MIC assay, strains were grown in MHB containing 0.2% arabinose to induce the expression of the gene of interest, and the strain harboring pMLBAD without any insert served as a control.

### Membrane potential measurement.

The measurement of the bacterial membrane potential followed previously described protocol (38). Briefly, mid-log-phase cultures were diluted to an OD_600_ of 0.2. Afterwards, 1 mL of the culture was added to 1.5 mL Eppendorf tubes and pelleted at 4,000 × *g* for 5 min. The pellets were resuspended in 500 μL 1× PBS containing 30 μM DiOC_2_ and were incubated at 37°C for 30 min. The bacterial suspensions were then pelleted, washed twice with 1× PBS, and resuspended in 1× PBS for measurement in a 96-well, black-sided, clear-bottom plate using a Tecan M200 Pro (Ex./Em. [Green]: 488/530; Ex./Em. [Red]: 488/630). The membrane potential was reported as a ratio of red over green fluorescence.

### Isothermal titration calorimetry.

The isothermal titration calorimetry experiment was performed in 5 mM Tris (pH 7.4) and 15 mM NaCl at 25°C using MicroCal PEAQ-ITC. Typically, 260 μL of 1 mM oligonucleotides (52 bp) that were loaded into a calorimetric cell using a syringe were titrated against a 1 mM OIM1-6 solution (19 injections of 2 μL each, with an initial injection of 0.4 μL) using a 40 μL syringe, rotating at 750 rpm. The titration of OIM1-6 solution into buffer served as a control. The resulting thermography was analyzed using a built-in, single binding sites model in the MicroCal analysis software package, and the respective dissociating constant *K_D_* as well as thermodynamic parameters were obtained.

### Molecular simulations of the binding of OIM1-6 to DNA.

Both DNA (the topoisomerase I/DNA complex [PDB ID: 1A36] [39] with the sequence of 5′-AAAAAGACTTAGAAAAATTTTT-3′) and OIM1-6 were constructed *in silico* using the CHARMM-GUI tool (40). The parameters of the DNA were based on the CHARMM36 force field (41), and the parameters of OIM1-6 were based on the CHARMM General Force Field (42). They were set 1 nm away from each other in the initial conditions. All of the topological files were converted to the GROMACS (43) format using the CHARMM-GUI force field converter. The system was solvated with TIP3P (44) water molecules, and counterions were added to neutralize the system. A molecular dynamics simulation was performed using the GROMACS 5.1.2 software package (43). The LINCS (45) algorithm was used to constrain bonds between heavy atoms and hydrogen to enable a timestep of 2 fs. A 1.2 nm cutoff was used for the calculations of Van der Waals interactions and short-range electrostatic interactions, and the particle mesh Ewald method was implemented for long range electrostatic calculations. The simulation temperature was maintained at 300 K, using a V-rescale thermostat and 1 bar pressure, using the Parrinello-Rahman barostat algorithm.

### Confocal microscopy.

Mid-log-phase cultures were adjusted to 10^6^ CFU/mL in MHB containing 4 μg/mL OIM1-6-Rho. For those with colistin cotreatment, 0.5× MIC of colistin was added to the bacterial samples. The bacteria were then incubated at 37°C for an hour before being pelleted, washed twice in 1× PBS and fixed with 1× PBS containing 1% paraformaldehyde at 4°C overnight. The fixed cells were washed with 1× PBS before they were transferred to poly-lysine-coated, 8-well, chambered slides (Thermo Fisher) for imaging with an FV3000 Olympus Confocal Microscope at ×100 magnification.

For the visualization of the dsDNA breaks using GamGFP, the E. coli MG1655 GamGFP strain (SMR14334) (17) was prepared, according to a previously described method (18). Briefly, an overnight culture of the strain was diluted at 1:100 in MHB for incubation at 37°C with shaking at 150 rpm. After an hour, a 20 ng/μL final concentration of doxycycline was added to induce the expression of GamGFP. The bacterial culture was grown to the mid-log phase and adjusted to 10^6^ CFU/mL in MHB. The cells were challenged with 10 mM hydrogen peroxide and 1× MIC OIM1-6 for 2 h. Then, the cells were fixed with 4% paraformaldehyde for 1 h and stained with 10 μg/mL Hoechst, prior to visualization with an FV3000 Olympus Confocal Microscope. The GamGFP foci of the cells were manually counted from the images and tabulated as percentage of the cells with foci for each treatment group.

### Flow cytometry.

The mid-log phase of the E. coli culture was diluted to 10^6^ CFU/mL in MHB. The bacterial suspension was mixed with OIM1-6 at a final concentration of 4, 8, or 16 μg/mL, and this was followed by 1 h of incubation at 37°C. The samples were pelleted at 4,000 × *g* and were washed twice with 1× PBS. Then, fixation with 1% paraformaldehyde in 1× PBS was performed overnight at 4°C. The solution was removed, and the cells were stained with picogreen at a 1:500 dilution for 1 h for analysis using a BD LSRFortessa Flow Cytometer. The picogreen signal was acquired under the Alexa Fluor 488 channel.

### Analysis of gene expression via real-time PCR.

The total bacterial RNA was isolated using TRIzol (Invitrogen) and a PureLink RNA Minikit (Invitrogen). cDNA was synthesized using a RevertAid First Strand cDNA Synthesis Kit (Thermo Fisher). Quantification was performed using iQ SYBR Green Supermix (Bio-Rad) in a CFX Connect real-time PCR detection system (Bio-Rad). The primers that were used are listed in Table S1. The relative mRNA levels were normalized to untreated cells using the threshold cycle (2^−ΔΔ^Ct) method (46), using *16S* as the reference gene.

### Statistical analyses.

The statistical analyses were performed using the analysis functions that were built into GraphPad Prism 8. Statistical significance was determined using unpaired *t* tests.
