# Supplementary material for: Membrane Potential-Dependent Uptake of Cationic Oligoimidazolium Mediates Bacterial DNA Damage and Death
Source: Antimicrob Agents Chemother. 2023 May 1;67(5):e00355-23. doi: 10.1128/aac.00355-23 (PMC10190574; doi:10.1128/aac.00355-23)
Supplement: Supplemental file 1 — Supplemental material. Download aac.00355-23-s0001.pdf, PDF file, 1.2 MB [file aac.00355-23-s0001.pdf]

SUPPLEMENTARY FIGURES

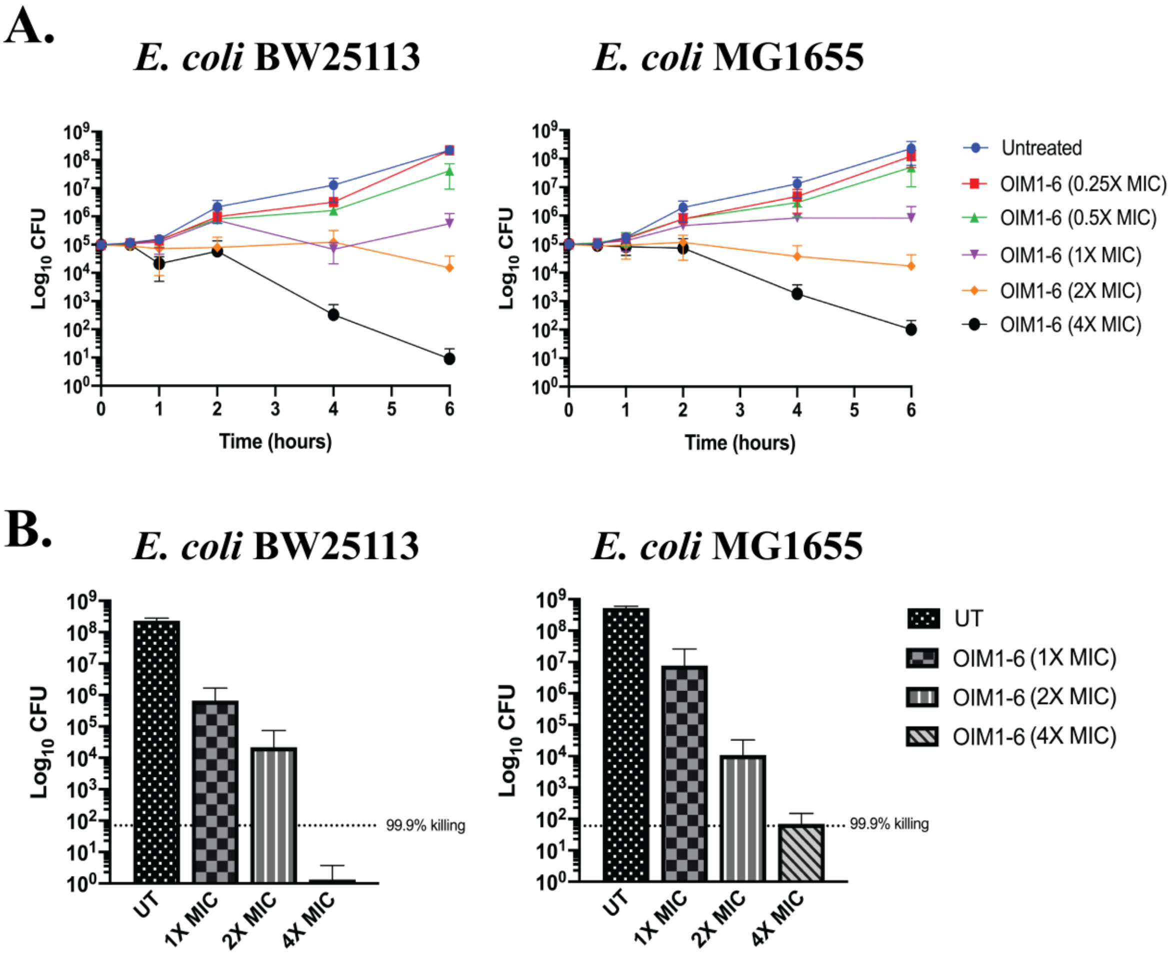

Fig. S1 Killing kinetics of OIM1-6 against *E. coli*. (A) Time kill assay of *E. coli* BW25113 and *E. coli* MG1655 challenged with OIM1-6. These strains were subjected to OIM1-6 at a concentration of 0.25X – 4X MIC for a duration of up to 6 hours. Bacterial cells were plated on LBA at specific timepoints to enumerate the viable bacteria which is expressed as colony forming unit (CFU). (B) Bacterial CFU after 24 hours of OIM1-6 treatment. The CFU was enumerated after 24 hours of incubation in absence or presence of OIM1-6 at 1 – 4X MIC concentration. Data shown is an average from three independent experiments. Error bars represent the standard deviation.

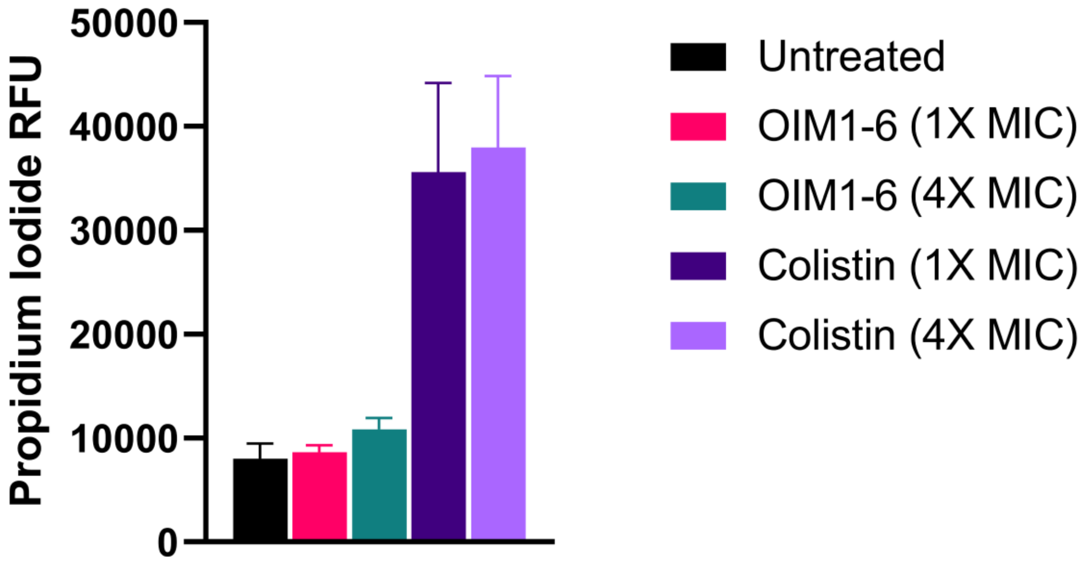

Fig. S2 Membrane integrity assay. *E. coli* MG1655 was treated with OIM1-6 or colistin at 1X and 4X MIC for an hour. Colistin serves as positive control for membrane disruption. The cells were stained with 15 μg/mL propidium iodide for 5 min before fluorescence intensity measurement at ex./em. 535nm and 615nm.

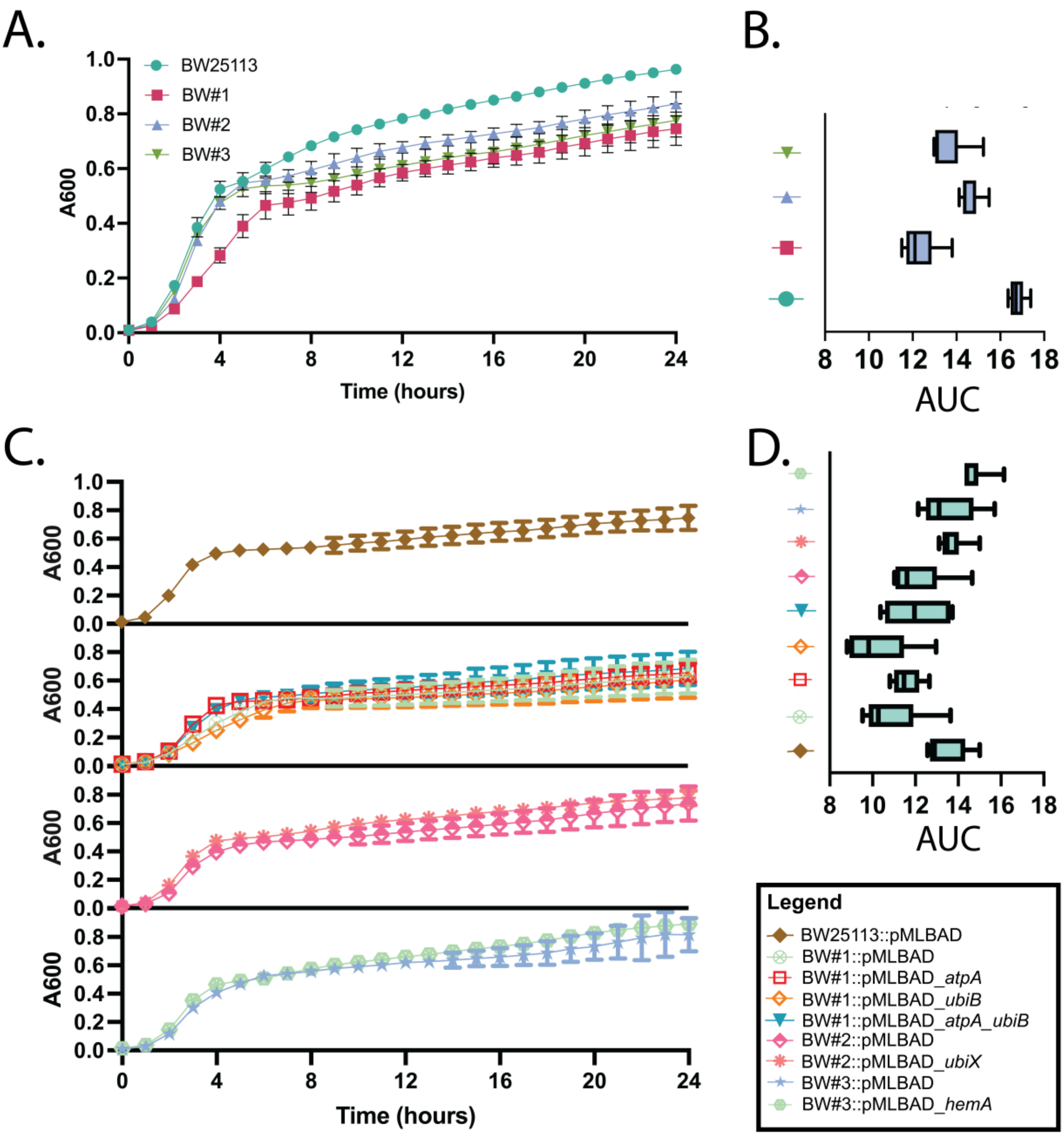

Fig. S3 Bacterial growth rate. (A) Growth rate of BW25113, BW#1, BW#2 and BW#3 grown in MHB at 37°C for 24 hours. (B) Area under the curve (AUC) derived from the growth curves shown in (A). (C) Growth rate of BW25113, BW#1, BW#2 and BW#3 carrying the empty pMLBAD plasmid or pMLBAD with a copy of the wildtype gene complementing their respective mutations. The growth rate of these strains were assessed in MHB supplemented with 0.2% arabinose for 24 hours at 37°C. (D) AUC derived from the growth curves shown in (C).

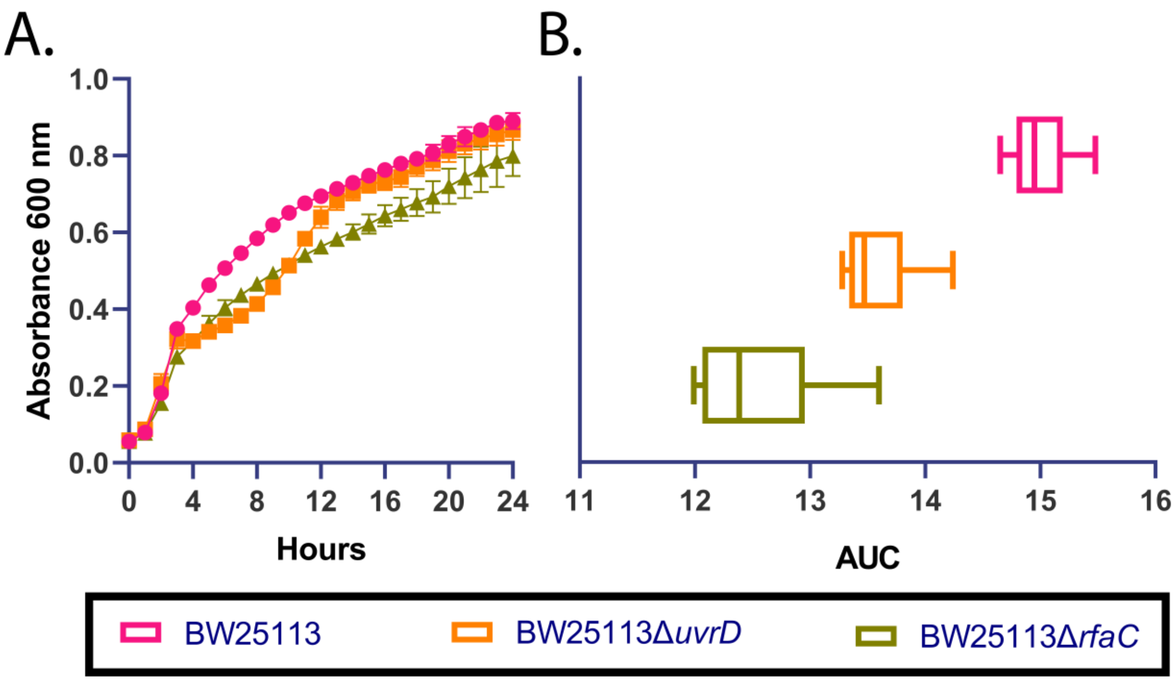

Fig. S4 Growth rate of BW25113 mutants. (A) Growth curve of BW25113, BW25113Δ*uvrD* (mutant of DNA helicase II) and BW25113Δ*rfaC* (mutant of lipopolysaccharide heptosyltransferase 1) grown in MHB for 24 hours at 37°C. (B) Area under the curve (AUC) of the growth curves shown in (A).

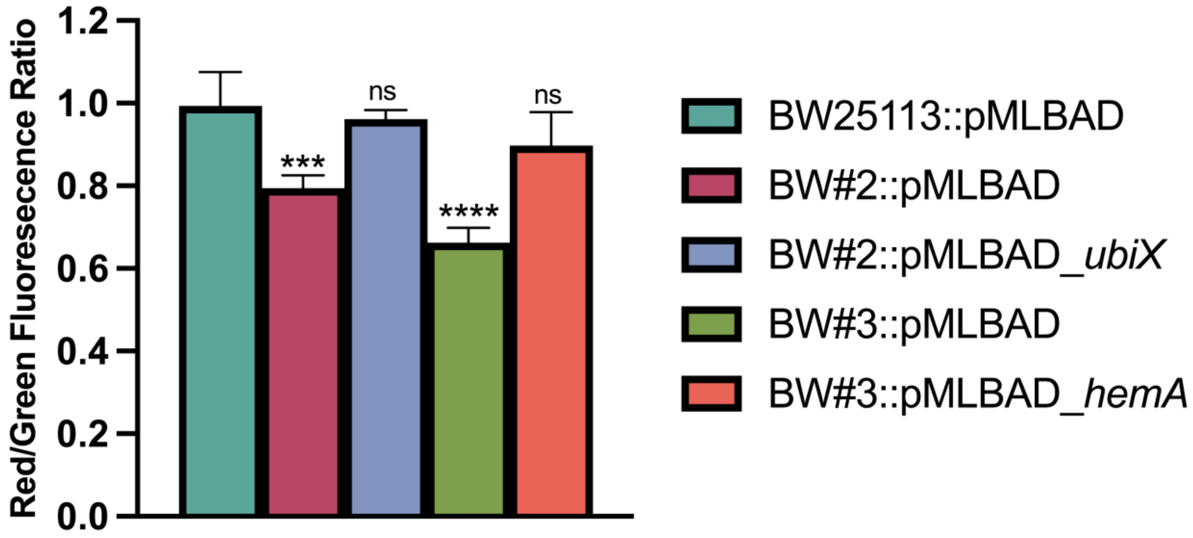

Fig. S5 Membrane potential measurement. Membrane potential of BW#2 and BW#3 with and without gene complementation. Error bars denote the standard deviation. Statistical significance between wildtype BW25113::pMLBAD and the other groups were assessed using unpaired t-test: ns, *p-value* > 0.05; ***, *p*-value ≤ 0.001; ****, *p-value* ≤ 0.0001.

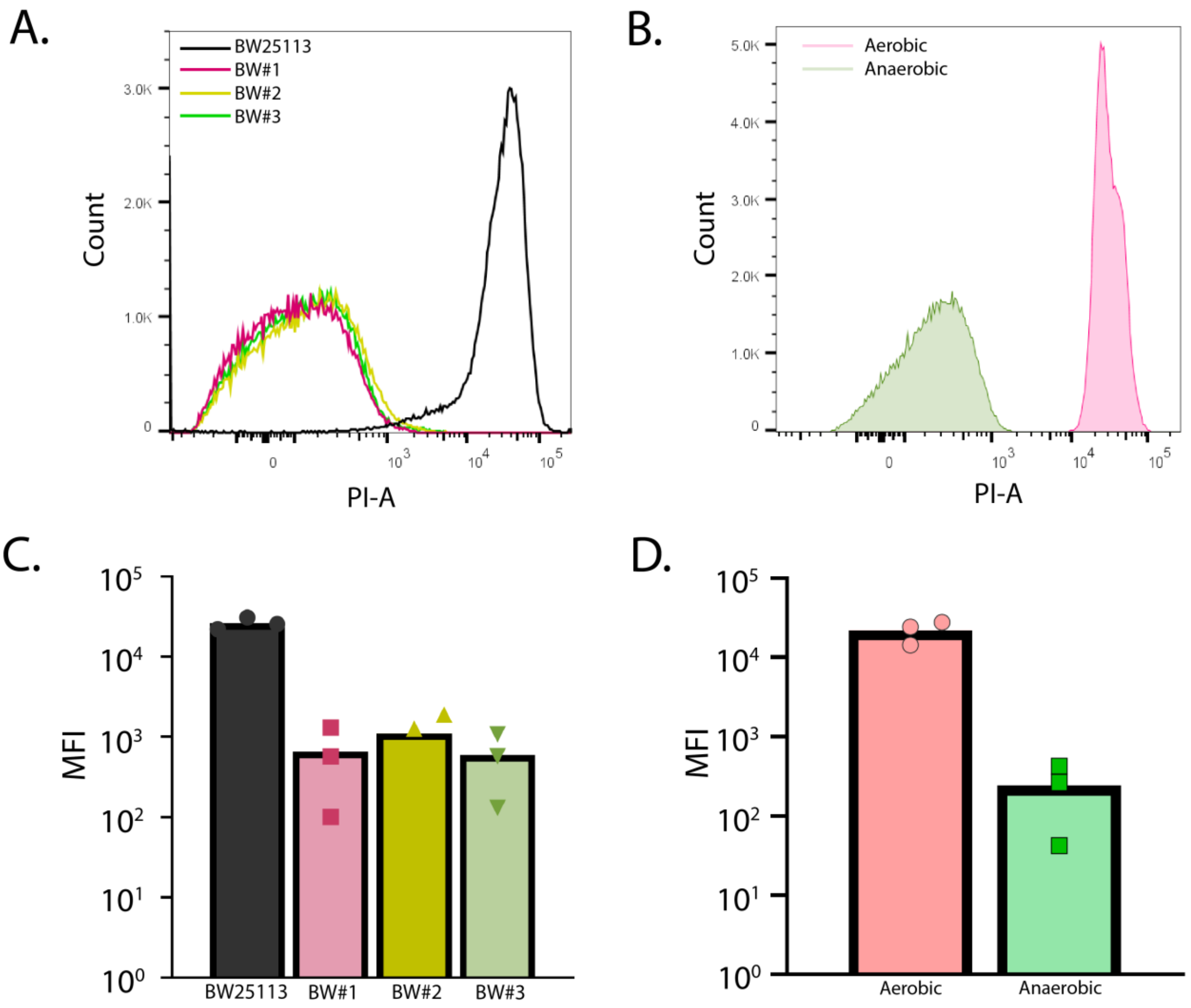

Fig. S6 Intracellular uptake of OIM1-6-Rho. (A) Intracellular uptake of OIM1-6-Rho in *E. coli* BW25113, BW#1, BW#2 and BW#3 and (B) aerobically and anaerobically grown *E. coli* MG1655 treated with 4 µg/mL OIM1-6-Rho for 1 hour in MHB at 37°C. These cells were subjected to flow cytometry analysis and the fluorescence intensity of OIM1-6-Rho was measured under PI channel. The median fluorescence intensity (MFI) of OIM1-6-Rho uptake from three independent experiments were tabulated in (C) for BW25113, BW#1, BW#2 and BW#3 and (D) for aerobic and anaerobic conditions.

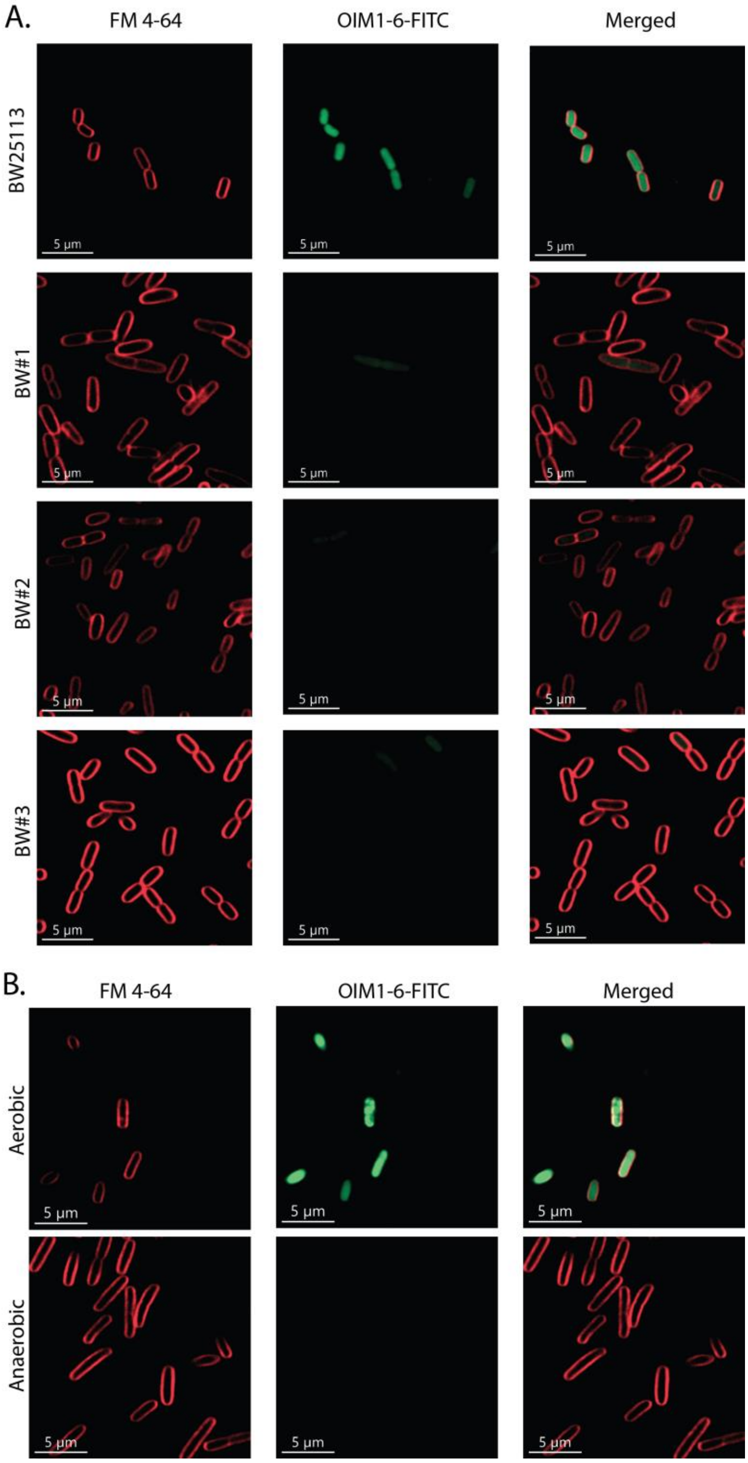

Fig. S7 Confocal microscopy using OIM1-6-FITC. (A) Representative confocal images of *E. coli* BW25113, BW#1, BW#2 and BW#3 treated with OIM1-6-FITC (1X $MIC_{BW25113}$) for an hour in MHB at 37°C. (B) Representative confocal images of *E. coli* MG1655 treated with OIM1-6-FITC (1X MIC_{MG1655}) for an hour in MHB at 37°C under aerobic and anaerobic growth conditions (anaerobic chamber). All the cells were stained with FM 4-64 dye for bacterial membrane visualisation.

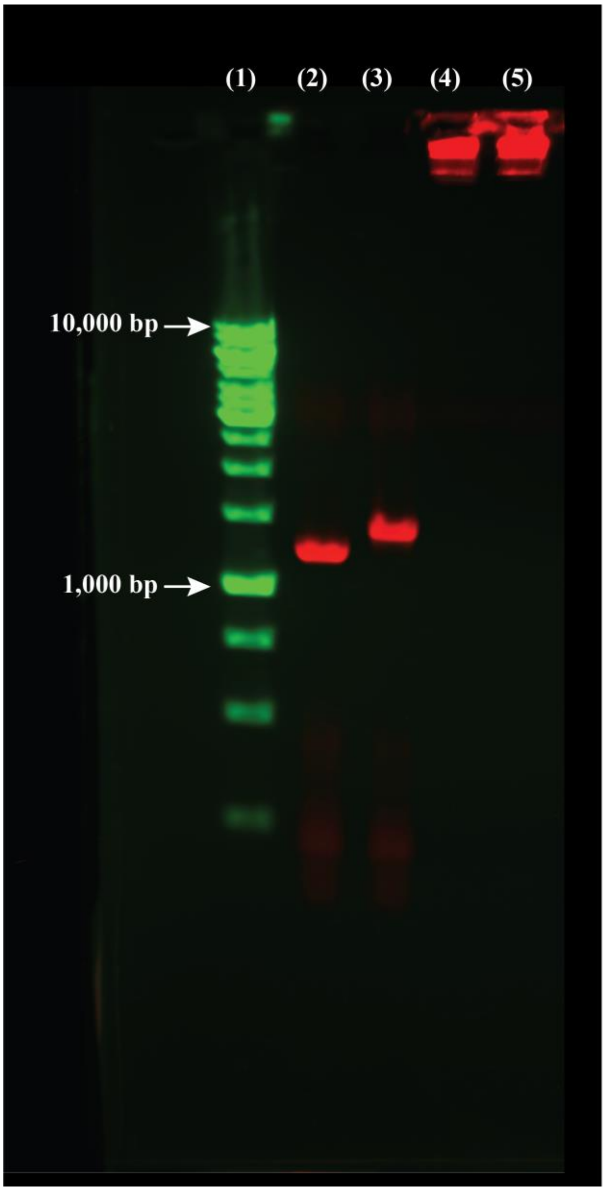

LEGEND

(1) Ladder
(2) DNA alone
(3) DNA + OIM1-6 (mass ratio = 10 : 1)
(4) DNA + OIM1-6 (mass ratio = 1 : 1)
(5) DNA + OIM1-6 (mass ratio = 1 : 10)

Fig. S8 Gel retardation assay to determine OIM1-6 interaction with DNA. Agarose gel electrophoresis using 100 ng Cy5-tagged DNA incubated for 10 min with OIM1-6 at mass ratio of 10:1, 1:1 and 1:10. The gel electrophoresis was performed on 1% agarose-TBE gel using 100 V for 60 min in 1X TBE buffer. Cy5-tagged DNA was prepared from PCR reaction using Cy5-tagged forward primer (/5Cy5/G AGTGATTAAACTCATACATTAACG TTTTC) and a non-fluorophore tagged reverse primer (AGTTGGTCTGGTGTCAAAAATA ATAATAAC) with purified *E. coli* MG1655 genomic DNA as template. The 1 kb PureLink DNA ladder (ThermoFisher) was stained with Quant-iT Picogreen for fluorescence imaging of the gel using ChemiDoc MP Imaging System (BioRad) with Alexa488 (for DNA ladder) and Cy5 (for Cy5-tagged DNA) filters.

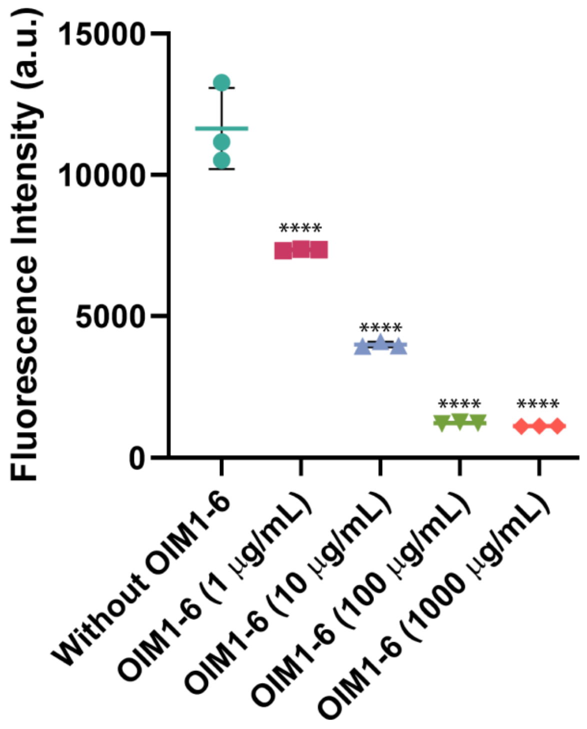

Fig. S9 In vitro picogreen displacement assay. 50 ng lambda DNA was prepared in the presence or absence of OIM1-6 at varying concentrations from 1-1000 μg/mL. Fluorescence intensity was measured using spectrophotometer (ex./em. = 480/520) after 10 min incubation at room temperature in a 96 well black polystyrene flat clear bottom plate (Greiner) with the addition of the Quant-iT Picogreen dye. Statistical significance between the control sample without OIM1-6 and the other groups were assessed using unpaired t-test: ****, *p-value* $\leq$ 0.0001.

SUPPLEMENTARY TABLES

Table S1. List of Primers

Primer Name	Primer Sequence
pMLBAD_atpA_F	CCATACCCGTTTTTTTGGGCTAGCAGGAGGTGC AGTCTTAAGGGGACTGG
pMLBAD_atpA_R	CGCCAAAACAGCCAAGCTTGCATGCCTGCAGTG ATCTTTTGCGTGTTCTGGAC
pMLBAD_ubiB_F	CCATACCCGTTTTTTTGGGCTAGCAGGAGGGCT TGAAGTGGCCTGGTTTGC
pMLBAD_ubiB_R	CGCCAAAACAGCCAAGCTTGCATGCCTGCAACG ATGACGGCAATAATCAATAACTG
pMLBAD_atpAubiB_p2	GTCTCTTCCGCAAACCAGGCCACTTCAAGCGTG ATCTTTTGCGTGTTCTGGAC
pMLBAD_atpAubiB_p3	CGCAAGCGTCCAGAACACGCAAAAGATCACGC TTGAAGTGGCCTGGTTTGC
pMLBAD_hemA_F	CCATACCCGTTTTTTTGGGCTAGCAGGAGGGCA TCCTGTATGATGCAAGCAGAC T
pMLBAD_hemA_R	CGCCAAAACAGCCAAGCTTGCATGCCTGCAAGC AACGCCTGAACTTCTTCA
pMLBAD_hemG_F	CCATACCCGTTTTTTTGGGCTAGCAGGAGGCAA CATGCTGTTTGGTCGTCT
pMLBAD_hemG_R	CGCCAAAACAGCCAAGCTTGCATGCCTGCACAG AGTCAAGCATTTATTTTTGCTTTCTCT
pMLBAD_hemL_F	CCATACCCGTTTTTTTGGGCTAGCAGGAGGCAC CAGTACAAGCAGCCTG
pMLBAD_hemL_R	CGCCAAAACAGCCAAGCTTGCATGCCTGCACCG AATCGTAGGTCGGATAAGGCGTTCA
pMLBAD_ubiX_F	CCATACCCGTTTTTTTGGGCTAGCAGGAGGTGC CGGTCTGGCACC
pMLBAD_ubiX_R	CGCCAAAACAGCCAAGCTTGCATGCCTGCAGCA GCGTTTCGATTGAATGGC
qPCR_16S_F	GGCTAGTCTAACCGCAAGGA
qPCR_16S_R	TCCGATACGGCTACCTTGTT
qPCR_recA_F	GGTACAGCTACAAAGGTGAG
qPCR_recA_R	GCCTTCGCTATCATCTACAG
qPCR_lexA_F	CTCATCCGTGATCACATCAG
qPCR_lexA_R	GCAACCCTTCTTCCTCTTC
qPCR_sulA_F	TGGGCTACCCTTAACGAA
qPCR_sulA_R	GCTTACCGGACGCATAATAA

Table S2. Minimum inhibitory concentration of gentamicin against OIM1-6 resistant strains

**Bacterial Strains**	**Minimum Inhibitory Concentration of Gentamicin (µg/mL)**
E. coli BW25113	0.5
E. coli BW#1	8
E. coli BW#2	4
E. coli BW#3	8
E. coli BW#4	4
E. coli BW#5	4
E. coli BW#6	8
E. coli BW#7	4
E. coli BW#8	8

Table S3. Minimum inhibitory concentration of rhodamine tagged OIM1-6

**Compounds**	**Minimum Inhibitory Concentration (µg/mL)**	
	***E. coli* MG1655**	***E. coli* BW25113**
**OIM1-6**	4	4
**OIM1-6-Rho**	4	4

Table S4. Minimum inhibitory concentration of OIM1-6 for anaerobically grown *E. coli*

**Bacterial Strains**	**Minimum Inhibitory Concentration (µg/mL) of OIM1-6**	
	**Aerobic**	**Anaerobic**
***E. coli* MG1655**	4	64
***E. coli* BW25113**	4	64
***E. coli* BW#1**	128	64
***E. coli* BW#2**	16	64-128
***E. coli* BW#3**	32	64-128
***E. coli* BW#4**	16	64-128
***E. coli* BW#5**	32	64-128
***E. coli* BW#6**	32	64-128
***E. coli* BW#7**	16	64
***E. coli* BW#8**	32	64-128
